# Conflicting Messages on Food and Beverage Packages: Front-of-Package Nutritional Labeling, Health and Nutrition Claims in Brazil

**DOI:** 10.3390/nu11122967

**Published:** 2019-12-05

**Authors:** Ana Clara Duran, Camila Zancheta Ricardo, Laís Amaral Mais, Ana Paula Bortoletto Martins, Lindsey Smith Taillie

**Affiliations:** 1 Center for Food Studies (NEPA), University of Campinas, Campinas 13083-852, Brazil; 2 Center for Epidemiological Studies in Nutrition and Health (NUPENS), University of Sao Paulo, Sao Paulo 01246-904, Brazil; ca.zancheta@gmail.com (C.Z.R.); anapaula@idec.org.br (A.P.B.M.); 3 Brazilian Institute for Consumers Defense (IDEC), Sao Paulo 05002-000, Brazil; lais.amaral@idec.org.br; 4 Department of Nutrition, University of North Carolina in Chapel Hill, Chapel Hill, NC 27516, USA; taillie@unc.edu

**Keywords:** claims, labeling, nutrition, consumer behavior, policy

## Abstract

We assessed the prevalence of front-of-package (FoP) claims in the Brazilian packaged food supply and examined whether foods with claims were more likely to be high in critical nutrients. Using data from a random 30% subsample of 11,434 foods and beverages collected in the five largest food retailers in Brazil in 2017 (*n* = 3491), we classified claims into nutrition, health, and environment. We examined whether foods with claims were more likely to be high in critical nutrients using 95% confidence intervals. Claims were found in 41.2% of the products. Nutrition claims were the most prevalent (28.5%), followed by health (22.1%), and environment-related claims (5.2%). More than 85% of the breakfast cereals, granola bars, and nectars contained claims, which were found in 51% of the dairy beverages. Foods with nutrition claims were more likely to be high in critical nutrients (65.3%; 95% CI 62.3, 68.2% vs. 54.1% 95% CI 52.1, 56.0). Products with health (52.9%; 95% CI 49.3, 56.4% vs. 58.5%; 95% CI 56.6, 60.3) and environment-related claims (33.5%; 95% CI 27.0, 40.8% vs. 58.6%; 95% CI 56.9, 60.2%) were less likely to be high in critical nutrients. FoP claims are prevalent in Brazil, and nutrition claims are more likely to have a poorer nutritional profile. Policymakers should consider restricting the presence of claims on unhealthy products.

## 1. Introduction

In recent decades, consumption of ultra-processed foods (UPF) in Brazil has rapidly increased [1], with current intake of these foods estimated to contribute 20–30% of a Brazilian’s daily caloric intake [2,3]. UPF, which are industrially formulated products usually containing additives, tend to have more sugar, saturated fat, and sodium; less fiber, and higher energy density [1]. Higher intake of UPF has also been associated with higher body mass index (BMI), increased odds of obesity, and excess weight in the Brazilian population [2,4]. In addition, a recent tightly controlled randomized trial found that UPF diets increased weight gain [5].

As part of a larger response to this increase in UPF in the Brazilian food supply, the Brazilian Health Regulatory Agency (Anvisa) is currently discussing the implementation of front-of-package (FoP) nutritional labeling in the country to help inform consumers about the nutritional content of packaged foods and beverages. Similar to the Chilean FoP warning label system, as well as systems adopted in Uruguay, Peru, and currently under final discussions in Mexico and Canada, the proposed labels in Brazil will be able to identify unhealthy foods only and would be applied to products containing excessive levels of added sugar, saturated fat, and sodium with the words “high in (sugar, saturated fat, or sodium)” [6]. Warning labels have been shown to be easier to understand than competing systems such as the traffic light among various groups, including Brazilians [7,8]. In the same proposal, Anvisa lays out options for restricting the use of nutrition claims in foods high in critical nutrients [9].

These proposed regulations have the potential to substantially alter the Brazilian food supply as well as consumers’ understanding, perceptions, purchase intentions, and intake of UPF. A recent randomized controlled trial in Brazil found that the exposure to FoP warning labels that only identify unhealthy foods helped consumers understand the unhealthiness of UPF and reduced their intentions to purchase this food [8], consistent with a growing body of literature that shows that warning labels improve consumers’ ability to assess the healthfulness of products [10,11] and help them make healthier choices [12,13,14,15]. Conversely, nutrition and health claims have been shown to make consumers think a product is healthier than it actually is, working as a marketing strategy rather than an information tool (i.e., the “health halo” effect) [16,17]. This body of research suggests that limiting the use of health and nutrition claims on products with FoP nutritional labeling could further improve consumers’ ability to assess the healthfulness of foods and beverages.

Thus, one important question is the prevalence of health and nutrition claims in the Brazilian food supply, and the proportion of these products that would carry FoP nutritional labeling under the proposed regulation. A related question is whether the nutritional profile of products with claims is more favorable compared with products without claims. Understanding the prevalence and nutritional profile of products which would be affected by the proposed regulation is important for developing the most effective policy to improve consumers’ ability to make healthier food decisions.

Considering much of the existing evidence is available for high-income countries [18,19,20,21,22], our paper contributes to the literature by showing the prevalence of nutrition, health, and environment-related claims in the food supply of a large middle-income country; and whether foods that carry those claims can be considered healthy.

The objectives of this study are to: (1) quantify the prevalence and type of front-of-package health, nutrition, and environment-related claims in the Brazilian food supply; and (2) examine the prevalence of foods high in critical nutrients and therefore eligible to receive front-of-package nutritional labeling on products with and without claims.

## 2. Methods

### 2.1. Database of Food Products Available in Brazilian Market

This was a cross-sectional study that took advantage of information from packaged foods and beverages sold in supermarket chains with the largest market share in Brazil in 2017. Supermarkets account for 59% of the calories purchased in the country [23], and the five supermarkets chains with the largest market share and that control 70% of the Brazilian market were identified using annual food retail sales organized by Euromonitor International in 2016 [24,25]. São Paulo, located in the Southeast region of the country, was chosen as the primary study area as it is the largest city in Brazil. Because one of the five top supermarket chains in Brazil only had stores in the Northeast region of the country, for that specific chain, data collection took place in Salvador, their largest market.

Data on the location of every store of the selected supermarkets chains in the cities of São Paulo and Salvador were gathered from each company’s website or customer service, and the addresses were geocoded. The neighborhood of each store was defined as a one-kilometer buffer (using Euclidean distance) around each store location. We used information on income from the household top earner from the latest Brazilian Census conducted in 2010 [26] to determine the mean neighborhood income around each store location. Stores were distributed in tertiles of neighborhood income, and the largest store in square meters of each chain in both the first and the third tertile was selected, except for one chain that only allowed us to collect data in its distribution center, where all the products sold in the stores are found. Formal permission to collect data was obtained from all the supermarkets chains included in this study.

Data were collected between April and July 2017 by trained fieldworkers, according to methods proposed by Kanter et al. (2017) [27]. All packaged foods and beverages were included, and around 13,000 different items had all sides of their package photographed. Data on composition information were entered between July and November 2017 by trained nutritionists in the online platform RedCap, using a template developed by the University of North Carolina at Chapel Hill (UNC) from the United States of America (USA) and by the *Instituto de Nutrición y Tecnología de los Alimentos* (INTA) from Chile, and adapted to be used in Brazil. In this stage, information collected included product information, package size, nutrition facts panel information, list of ingredients, and information about reconstitution (when applied). After the exclusion of items available in more than one package size, products without nutrition information, multipack with different items, products without a list of ingredients, and products with missing values for portion size and/or calories, 11,434 records were maintained in our database.

### 2.2. Claims Information and Classification

The taxonomy developed by The International Network for Food and Obesity/Non-communicable Diseases (NCDs) Research, Monitoring and Action Support (INFORMAS) was used to classify different types of claims featuring on food and beverages packages [28]. The INFORMAS taxonomy divides claims into three major categories: nutrition claims, health claims, and other claims, which includes other health-related claims and environment-related claims. In our study, we decided to combine other health-related claims with health claims. In addition, we used sub-categories for nutrition and health claims (Table 1).

All visible text was allowable to be coded as a claim, including brand names and slogans. We did not consider as a claim the mention of substances in the list of ingredients, and the mention of nutrients as a mandatory part of nutrition labeling. In the case of claims that could be classified as more than one type of claim, the following hierarchy was applied for classification: health claim, nutrition claim, and other claim. For this study, we only considered claims in the front of the package.

We gathered information available on the food labels on health, nutrition, and environmental claims in a random subsample of 30% of all the products food in the main supermarket chains in Brazil. A 30% random sample was drawn from each of the 128 categories of food primarily used in data entering, yielding 3491 products. We did not find any statistical differences in food composition when we compared this random sample with the universe of photographed food packages.

### 2.3. Classification of Products According to Their Nutritional Profile

The Pan American Health Organization (PAHO) nutrient profile model (NPM) was used to classify foods according to their nutritional profile because it was developed to be used in various food and nutrition policies in Latin America, including labeling, and identifies unhealthy foods being aligned with the proposed FoP regulations under discussion in Brazil [30]. This NPM considers the level and degree of industrial processing, according to the NOVA classification, as an eligibility criterion. It classifies food products as containing or not excessive amounts of five nutrients: free sugar, total fats, saturated fats, trans fat, and sodium. In addition, the presence of nonnutritive sweeteners in the list of ingredients is also considered in the model. The thresholds determined in the model are applied on the ratio between the content of critical nutrients and the content of energy, and is based on the World Health Organization (WHO) recommendations to prevent obesity and chronic diseases [30]. In our study, we included a modification to the original PAHO NPM, in which a food or beverage was eligible to be regulated and therefore receive FOP warning signs based on the Chilean nutritional labeling law eligibility criteria (Law 20.606/2015). In the Chilean law, only foods and beverages with added salt, sugar, or saturated fat were eligible to receive FOP warning signs for “high in” critical nutrients [31]. Culinary ingredients, as sugar, salt, oils, butter, and milk creams were only included if the product had the addition of another critical nutrient in excessive amounts (for instance, butter made with milk cream and salt is eligible to be regulated and receives a warning sign for high content of sodium if this nutrient is in excess—however, it does not receive a warning sign related to the high content of fats). For this modified PAHO NPM, we used the same targeted five nutrients (free sugar, total fats, saturated fats, trans fat, and sodium) as well as nonnutritive sweeteners and applied the same threshold levels as the PAHO NPM, and the model behaved similarly to the originally proposed PAHO NPM in terms of identifying foods high in critical nutrients [32].

The PAHO NPM considers free sugars, information that is not available on food labels of products sold in Brazil. We thus estimated the amount of free sugars using the method proposed by PAHO that considers the information on the amount of total sugars declared on food labels [30]. In this method, foods are classified by the information available on the nutrition facts panel (total sugars or added sugars) and by food category. For instance, if information on total sugars is available and the product has no or a minimal amount of naturally occurring sugars, such as soda and sports drinks, then the total amount of added sugars is considered free sugars. For milk or yogurt with any type of sugar in the list of ingredients, 50% of the declared added sugars were considered free sugars, so lactose, galactose, and other types of naturally occurring sugars were less likely to be considered free sugars. In Brazil, the content of total sugar is not required to be present on nutrition facts panel in the country, and analyses that considered free sugars were conducted for 10% of the sample that provided this information.

In order to apply the PAHO nutrient profile model, products were classified as containing added sugar, sodium, fat, and nonnutritive sweeteners on the basis of keyword searches in the list of ingredients. Briefly, ingredients used as a proxy for added sugars included sugar, honey, syrups, molasses, maltodextrin, glucose, fructose, and concentrated fruit and vegetables juices, as well as chocolate and milk fondant. Ingredients for the addition of salt included salt, sodium chloride, cheeses, and processed meats. For fat, we considered oils, olives, butter, creams, and animal and vegetal fats. Nonnutritive sweeteners included aspartame, saccharin, sucralose, cyclamate, acesulfame k, stevia, polydextrose, maltitol, mannitol, isomaltose, neotame, xylitol, thaumatin, and advantame. All searches were made in Portuguese.

### 2.4. Reliability Analysis

To assess inter-rater and test–retest reliability of food composition data, 10% of the foods were double-entered. We used the intraclass coefficient to assess inter and test–retest reliability and found strong inter-rater and test-retest reliability (ICC ≥ 0.90) for all assessed nutrients [33].

Information on claims was entered twice for all 3491 products. We used Cohen’s kappa to assess inter-rater and test–rest reliability of the claims’ entered information. Considering that coefficients above 0.80 are considered to show a strong to an almost perfect agreement [34,35], we found strong reliability for all assessed claims (Cohen’s kappa ≥ 0.82), except for claims related to the reduction of disease risk, which had coefficients ranging from 0.55 to 0.76.

### 2.5. Analysis

We first assessed the prevalence of health, nutrition, and environment-related claims in the front-of-package overall, and by food categories. Then, considering the current discussions on the Brazilian nutritional labeling regulatory process [9], that, among other things, includes requirements for FoP nutritional labeling, we first described how many foods with FoP claims were high in critical nutrients and therefore eligible to receive FoP nutritional labeling. Thirdly, we examined whether foods and beverages with different types of claims were more likely to be high in critical nutrients using 95% confidence intervals. We checked whether different types of nutrition claims (for nutrients of concern or not of concern) and health claims (general health claims, claims for special diets, and for natural foods) were more likely to be high in critical nutrients using 95% confidence intervals. Finally, we present stratified analysis of the proportion of different types of claims by foods high in free sugars, saturated fat, and sodium.

## 3. Results

**Figure 1:**
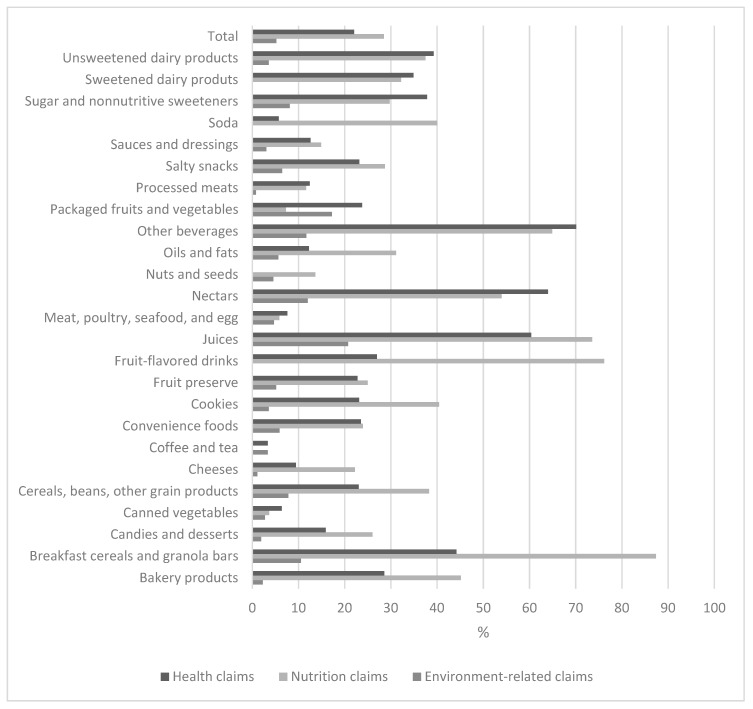
Presence of claims (%) in the front of the package of Brazilian packaged foods and beverages, 2017.

Figure 1 shows the presence of claims in the front of the package of foods and beverages by food categories. Overall, 41.2% of the assessed products presented claims, however, in some categories, claims were present in more than 80% of the products, such as breakfast cereals and granola bars (93.7%), fruit juices and nectars (92.5%), and fruit-flavored drinks (84.1%). Nutrition claims were more frequently found in Brazilian packaged foods and beverages (28.5%), followed by health claims (22.1%). Although environment-related claims were found to be used less frequently in packaged foods and beverages (5.2%), within fruit juices and packaged fruits and vegetables, around 20% of the products presented such claims. We found nutrition and health claims in more than a third of sweetened and unsweetened dairy products and in over 50% of juices, nectars, and other sweetened beverages such as those plant-based beverages. Nutrition claims were found in 40.0% of carbonated beverages. Among cookies, 40.4% and 23.1% of them had nutrition and health claims, respectively; and 87.4% of the breakfast cereals and granola bars had nutrition claims, while 44.2% had health claims.

**Figure 2:**
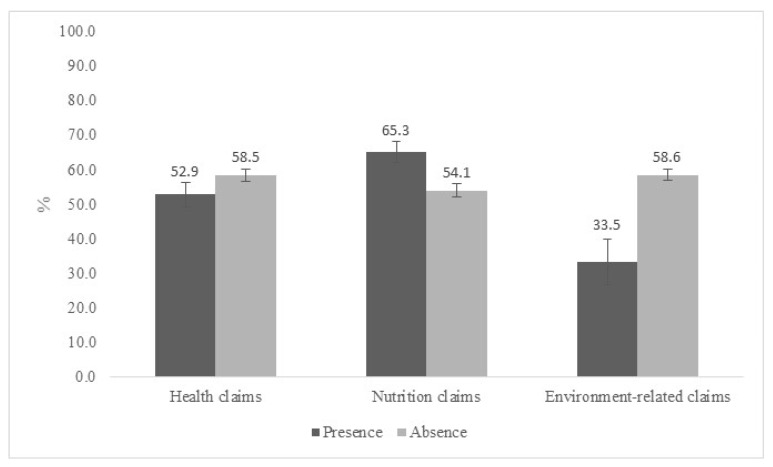
Presence of high level of critical nutrients according to front-of-package claims in Brazilian packaged foods and beverages, 2017. Note: Bars denote 95% confidence intervals.

We found claims in a quarter (23.5%) of the assessed products that were high in any of the critical nutrients according to the PAHO NPM. The percentages of foods and beverages high in any of the assessed critical nutrients (free sugar, total fats, saturated fats, trans fat, and sodium) among those with or without claims in the front of the package are shown in Figure 2. More products with nutrition claims (65.3%; 95% CI 62.3, 68.2% vs. 54.1% 95% CI 52.1, 56.0) were high in critical nutrients, and therefore eligible to receive FoP warning signs, as compared with those not eligible to receive FoP warning signs. On the other hand, fewer products with health claims (52.9%; 95% CI 49.3–56.4% vs. 58.5%; 95% CI 56.6, 60.3) and environment-related claims (33.5%; 95% CI 27.0, 40.8% vs. 58.6%; 95% CI 56.9, 60.2%) were high in critical nutrients.

Table 2 shows the foods and beverages high in critical nutrients among those with or without claims in the front of the package by food categories. Among the products presenting health claims, salty snacks, candies and desserts, and fruit-flavored drinks had fewer products high in critical nutrients. More sweetened dairy products high in critical nutrients were found among those with nutrition claims. For fruit-flavored drinks and soda, we found similar results: of those with nutrition claims, more than 80% were high in critical nutrients as compared with 46.7% and 33.3% of those without claims, respectively. Of the foods with information for the content of free sugars and claims, 38.6% had health claims and 44.4% had nutrition claims. Similarly, of the foods high in sodium, a quarter (24.5%) had health claims and a third (29.2%) had nutrition claims. Claims were slightly less present in foods high in saturated fat (18–21%) (Table S1 in Supplementary Materials).

We show in Table 3 and Table 4 findings for the subcategories of health and nutrition claims, respectively. General health claims were found in 8.5% (95% CI 7.6, 9.5) of the assessed products. Claims for special diets and “natural” were found in 7.0% (95% CI 6.2, 7.9), and 9.0% (95% CI 8.1, 10.0) of the products, respectively. Among products with claims for “natural”, fewer products (43.5%; 95% CI 38.3, 48.9 vs. 58.7%; 95% CI 57.0, 60.4) were high in critical nutrients. The proportion of foods with general health claims and claims for special diets was similar across products with and without high levels of critical nutrients.

Nutrition claims were divided in claims for nutrients of concern—present in 12.7% of the assessed products and that have been associated with increased cardiovascular disease risk—and not of concern (present in 13.1% of the assessed products) (Table 4). Of foods and beverages with claims for sodium, sugar, saturated fat, and trans-fat (of concern), three quarters were high in critical nutrients—a larger proportion when compared with foods that were not high in critical nutrients (74.0%; 95% CI 69.7, 77.9 vs. 54.8%; 95% CI 53.0, 56.6). Such differences were not found for claims for nutrients that were considered not of concern.

## 4. Discussion

Our study provides an overview of the extent and nature of nutrition and health claims on the FoP in packaged foods and beverages sold in the Brazilian largest retailers. Overall, 41% of the products featured claims on the FoP, indicating extensive use of nutrition, health, and to a lesser extent environment-related claims on packaged foods sold in Brazil. We found claims in a quarter (23.5%) of the assessed products that were high in any of the critical nutrients according to the PAHO NPM; and that more foods and beverages with nutrition claims were high in critical nutrients, as compared with those low in critical nutrients.

In other words, should FoP nutritional labeling be implemented on foods high in critical nutrients in Brazil, more products with such labeling would have more nutrition claim than products without FoP nutritional labeling if restrictions for the presence of nutrition claims in foods high in critical nutrients are not implemented concomitantly. These products would thus have conflicting messages in the front-of-package. On the other hand, we found that fewer products with health claims and environment-related claims were high in critical nutrients.

Nutrition claims were indeed the most prevalent type of claim found in the Brazilian packaged food supply, covering almost a third of the assessed products as found in other countries [19,20,21,36,37]. Health claims were also quite prevalent and were found in 22% of the assessed products. The proportion of claims found in some of the food categories, particularly those with a high consumption among children and adolescents in the country, such as sweetened dairy and non-dairy beverages [38,39], were even higher. Nutrition claims were found in 32% of the sweetened dairy products, whereas health claims were found in 34% of the same products. In a study conducted in five European countries, nutrition and/or health claims were found in 46% of the dairy products—the largest proportion among the studied products [19].

Nutrition and health claims were also found in more than half of most types of sweetened beverages, such as juices, nectars, carbonated beverages, and other beverages (i.e., plant-based beverages). Considering sweetened beverages contribute with 49% of the added sugars consumption in Brazil [38], allowing such foods to carry health and nutrition claims can jeopardize the ability of consumers to make informed healthier choices and contribute to increasing their consumption of added sugars. Such issue could particularly impact the consumption of added sugars among Brazilian children and adolescents. For example, a third of Brazilian children under the age of two consume sweetened beverages [40], and adolescents report sweetened dairy beverages among the most consumed food items [41]. In fact, the proportional contribution of sweetened dairy beverages to added sugars intake among adolescents is higher than that found for adults and elders [38].

Forty percent of the assessed cookies and 29% of the salty snacks, two other highly consumed items among adolescents in Brazil [2], had nutrition claims, comparable to previously found in a smaller sample from Brazil [37]. Interestingly, of the 16 assessed countries in the same survey, the authors found the second largest proportion of nutrition claims among cookies and chips sold in Brazil (50%) [37]. The prevalence of marketing geared towards children in these same foods sold in Brazil was also among the highest found in the same survey [37]. Another study conducted in one single Brazilian supermarket found that half of the products marketed towards children bore nutrient claims and 95% of them were classified as ultra-processed foods [42].

Additionally, we found that more foods with nutrition claims—more specifically, those foods with claims for nutrients considered of concern—that have been associated with increased cardiovascular disease risk, such as saturated fat, trans fats, sugar, and sodium [29,30], were high in critical nutrients when compared with foods without such claims. On the other hand, we did not find any difference in foods with presence of nutrition claims for other nutrients (e.g., vitamins, minerals, unsaturated fats). Future analyses could consider looking more closely at nutritional claim sub-types (e.g., presence of beneficial nutrients such as Vitamin C vs. absence of nutrients of concern, such as low sugar) to better understand the link between claims and the nutritional profile of products.

Although by a small margin, more products with health claims had a better nutritional profile that those without such claims, as found elsewhere [19,20,21]. When we looked at subcategories of health claims, only products with health claims for “natural” were less likely to be high in critical nutrients, which may have driven the overall small difference in foods with health claims by the presence of high levels of critical nutrients.

Taken together, our findings from a large sample of Brazilian packaged foods and beverages, sold in supermarkets in the country that control 70% of the retail market share contribute to the literature by showing the pervasive presence of nutrition and health claims in unhealthy products, even more so among those highly consumed by children and adolescents [2,25,40]. Although FoP labeling in the format of warning signs seems to be effective in nudging consumers to choose healthier products [8,11,14,43], when these signs are accompanied by nutrition claims for the same nutrient for which a warning sign is present in the package, this can undermine the efficacy of FoP labeling [17]. Health and nutrition claims have been associated with purchase behavior [44]. Nonetheless, the misleading nature of nutrition claims has been depicted in previous studies [45]. Groups with special dietary needs as well as those with illnesses and parents refer to be more likely to benefit from health and nutrition claims and consider that overall nutrition and health claims made them more interested in a product which they considered to be healthy [46]. Restricting, thus, the presence of claims—particularly nutrition claims in foods high in critical nutrients—should be part of regulatory processes that aim to help consumers make healthier choices at the point of purchase.

Less information in the literature is found for environment-related claims. In our study, claims such as organic, biodiversity, and genetically modified, organism free were classified as being environmentally related. Other studies may have included them as part of health claims. We found these specific claims in a smaller proportion of products (5%) as compared with health and nutrition claims, but a fifth of fruit juices and packaged fruits and vegetables depicted them. Fewer foods and beverages with environment-related claims were high in any of the assessed critical nutrients (free sugar, total fats, saturated fats, trans fat, and sodium).

Claims for “organic” are associated with the food production method, but this claim seems to represent a cluster of attributes that goes beyond production-specific characteristics (e.g., pest management, fertilizer usage, and soil treatment). Organic products or other “eco-friendly” foods are associated with ethical, health, and environmental concerns, as well as nutrition and food safety aspects. They have been shown to be higher in antioxidants and reduce consumers’ exposure to pesticides more than their conventional counterparts [47]. Nonetheless, an “eco-friendly” or “organic” claim can lure people into believing it is healthier than an alternative, when this may not be true [48]. Consumers can infer proprieties that are unrelated to the production method, perceiving organic foods as healthier, tastier, and less caloric than those produced conventionally [49]. Perceived healthfulness of a food product and use of food labeling, in turn, influences food intake [50].

Our study has a few limitations. First, we used a random subsample to evaluate claims, which may have introduced bias in our results. Nonetheless, we were cautious in selecting our subsample, which did not statistically differ from the total packaged food supply sample in terms of the proportion of foods in each food category or on the average content of nutrients. Second, we did not weigh the products by market share and did not specifically consider the most consumed foods. However, we included a considerable sample (over 10,000 items) of foods sold by the five top grocery retailers in Brazil. Third, we only included packaged foods that have information on the ingredients list and a nutrition facts panel in our sample, however, in Brazil, all packaged foods are required to depict the ingredients list and the nutrition facts panel in their label. Forth, information on the content of total sugar is not required to be present in the nutrition facts panel in Brazil. Analyses that considered total or free sugars were conducted in 10% of the sample that provided information for the content of total sugars in the nutrition facts panel, which may have underestimated the number of products that would be required to receive FoP labeling for high content of sugar. The method we used to estimate free sugars may have also introduced bias in the proportion of foods classified as high in sugar. The method we used classifies all foods and beverages in only four categories and defines the proportion of total sugar that should be considered as free sugars (zero, 50%, 75%, or 100%) based on food groups. This method is accurate for products without any free sugar and for those in which all the sugar comes from a source of free sugar. However, for dairy and fruit-based products such as fruit preserves and juices, for example, that have added and intrinsic sugars, the estimation could be biased. Although a few other methods to estimate free sugars are available, no method is considered standard, free of limitations, and applicable to all contexts [51,52].

Strengths of our paper include our sample that draws from all available packaged foods and beverages found in the five largest food retailers in the country, and the taxonomy we used to classify claims proposed by INFORMAS for which we found strong inter-rater reliability. This standardized classification of health-related labelling components has been used in different countries and therefore allows for international comparison [28].

In conclusion, we found a pervasive presence of health and nutrition claims in the Brazilian food supply and a larger proportion of nutrition claims than other types of claims. Moreover, a quarter of the Brazilian food supply that carries a claim was found to be high in critical nutrients and would therefore receive FoP nutritional labeling should a FoP nutritional labeling system be implemented in Brazil. Additionally, we found that more foods with nutrition claims—in particular, those foods with claims for nutrients that have been associated with increased cardiovascular disease risk—were high in critical nutrients when compared with foods without such claims. Restricting the presence of nutrition claims in foods high in critical nutrients should be part of regulatory processes that aim to help consumers make healthier choices at the point of purchase and an essential part of discussions to implement clearer FoP nutritional labeling.

## Figures and Tables

**Table 1:** Categories of claims.

Categories of Claims	Subcategories of Claims	Content of Claims
Health Claims	General health claims	Claims related to general beneficial health effects (e.g., healthy, fit, nutritious).
	Claims for special diets ^1^	Claims related with allergies/intolerance (e.g., gluten free, dairy free), vegetarian/vegan.
	Claims for ‘Natural’ ^1^	Claims related to natural/pure products, and absence of additives, pesticides, and hormones.
	Others	Nutrient and function claims, reduction of disease risk claims.
Nutrition Claims	Nutrients of concern ^2^	Nutrient comparative or nutrient content claims related to saturated fat, trans fat, sugar, and sodium.
	Nutrients not of concern	Nutrient comparative or nutrient content claims related to fiber, vitamins and minerals, unsaturated fatty acids, and protein.
	Others	Health-related ingredients (e.g., quantity of fruits, nuts, wholegrains), claims related to nutrients not included in the other sub-categories created (e.g., carbohydrates).
Environment-Related Claims		Organic, biodiversity, genetically modified organism free.

^1^ Although special diets and natural-related claims are classified as “Other Claims” in the INFORMAS taxonomy, we combined them with the other health-related claims; ^2^ We considered nutrients of concern those for which excess intake is associated with increased non-communicable disease risk [29].

**Table 2:** Foods and beverages high in critical nutrient by the presence of different types of claims in the front of the package, Brazil, 2017.

Food Category	Health Claims						Nutrition Claims						Environment-Related Claims					
	Yes			No			Yes			No			Yes			No		
	%	95% CI		%	95% CI		%	95% CI		%	95% CI		%	95% CI		%	95% CI	
Breakfast Cereals and Granola Bars	57.1	41.8	71.2	62.3	48.5	74.3	61.4	50.5	71.3	50.0	23.5	76.5	50.0	21.2	78.8	61.2	50.4	71.0
Bakery Products	92.0	80.4	97.0	87.2	80.1	92.0	91.1	82.5	95.7	86.5	78.0	92.0	50.0	9.3	90.7	89.5	83.9	93.3
Convenience Foods	83.9	71.8	91.5	90.7	85.47	94.12	87.7	76.3	94.1	89.5	84.1	93.2	85.7	55.7	96.6	89.3	84.5	92.7
Unsweetened Dairy Products	4.5	0.6	27.1	0.0	.	.	4.8	0.6	28.2	0.0	.	.	0.0	.	.	1.9	0.3	12.2
Sweetened Dairy Products	48.1	34.8	61.6	43.3	33.8	53.4	77.1	63.0	86.9	29.7	21.6	39.4	0.0	.	.	45.0	37.1	53.0
Salty Snacks	76.0	55.3	89.0	96.4	89.3	98.8	83.9	66.3	93.2	94.8	86.9	98.1	100.0	.	.	91.1	83.7	95.3
Cookies	92.3	81.1	97.1	92.5	87.5	95.6	92.3	84.7	96.3	92.5	86.7	95.9	100.0	.	.	92.2	87.7	95.1
Canned Vegetables	71.4	29.8	93.6	86.4	78.3	91.8	75.0	18.0	97.6	85.8	77.8	91.3	100.0	.	.	85.0	76.9	90.7
Oils and Fats	30.8	11.5	60.3	12.9	7.5	21.4	36.4	21.7	54.0	5.5	2.1	13.8	0.0	.	.	16.0	10.0	24.6
Sauces and Dressings	75.8	58.2	87.5	85.6	80.4	89.6	92.3	78.4	97.5	83.0	77.4	87.4	62.5	26.3	88.6	85.0	80.1	88.9
Coffee and Tea	0.0	.	.	0.0	.	.	0.0	.	.	0.0	.	.	0.0	.	.	0.0	.	.
Candies and Desserts	0.0	0.0	0.0	71.7	66.4	76.4	78.9	69.5	86.0	69.3	63.5	74.5	57.1	20.7	87.2	72.1	67.2	76.5
Cereals, Beans, Other Grain Products	3.8	0.9	14.1	87.0	81.18	91.22	0.0	.	.	11.3	7.0	17.6	5.6	0.7	32.2	11.3	7.7	16.4
Packaged Fruits and Vegetables	5.5	1.7	15.8	0.0	.	.	0.0	.	.	0.0	.	.	0.0	.	.	0.0	.	.
Meat, Poultry, Seafood, and Egg	0.0	.	.	0.0	.	.	0.0	.	.	0.0	.	.	0.0	.	.	0.0	.	.
Sugar and Nonnutritive Sweeteners	0.0	.	.	21.7	9.2	43.4	90.9	53.6	98.9	15.4	5.8	35.0	0.0	.	.	41.2	25.9	58.3
Processed Meats	64.3	36.6	84.9	92.4	88.2	95.3	93.3	76.5	98.4	93.0	88.8	95.6	100.0	.	.	92.9	89.1	95.5
Juices	96.9	80.3	99.6	23.8	10.0	46.7	7.7	2.5	21.6	28.6	10.7	57.1	27.3	8.5	60.4	9.5	3.6	23.0
Nectars	6.3	1.5	22.2	27.8	11.7	52.7	29.6	15.3	49.4	21.7	9.2	43.4	16.7	1.8	68.1	27.3	16.1	42.3
Fruit-Flavored Drinks	25.0	12.9	42.9	87.0	73.7	94.1	87.5	74.7	94.3	46.7	23.4	71.4	0.0	.	.	77.8	65.8	86.4
Soda	52.9	29.6	75.0	54.5	37.4	70.7	92.9	61.1	99.1	33.3	16.5	55.9	0.0	.	.	57.1	40.3	72.5
Other Beverages	100.0	.	.	78.3	56.6	90.8	80.0	66.5	89.0	44.4	27.0	63.4	66.7	31.3	89.8	67.6	55.6	77.7
Nuts and Seeds	63.0	49.3	74.8	31.8	15.7	53.9	33.3	2.6	90.5	31.6	14.6	55.5	0.0	.	.	33.3	16.5	55.9
Cheese	82.4	56.3	94.4	91.4	86.0	94.9	92.5	78.9	97.6	90.0	83.8	94.0	100.0	.	.	90.4	85.2	94.0
Fruit Preserve	32.3	18.1	50.6	7.6	3.8	14.5	32.4	18.7	49.8	6.9	3.3	13.8	0.0	.	.	14.0	9.0	21.1

Abbreviation: CI, confidence interval.

**Table 3:** Foods and beverages high in critical nutrients by the presence of subcategories of health claims in the front of the package, Brazil, 2017.

Food Category	General Health Claims						Claims for Special Diets						Claims for “Natural”					
	Yes			No			Yes			No			Yes			No		
	%	95% CI		%	95% CI		%	95% CI		%	95% CI		%	95% CI		%	95% CI	
Breakfast Cereals and Granola Bars	50.0	30.6	69.4	63.4	51.6	73.8	66.7	31.3	89.8	59.3	48.6	69.2	53.8	27.2	78.5	61.0	50.0	70.9
Bakery Products	88.9	63.7	97.3	88.5	82.5	92.7	90.0	66.6	97.6	88.4	82.3	92.6	88.9	63.7	97.3	88.5	82.5	92.7
Convenience Foods	93.3	63.0	99.1	88.8	83.9	92.3	88.5	69.1	96.3	89.2	84.2	92.7	86.1	70.3	94.2	89.6	84.6	93.1
Unsweetened Dairy Products	0.0	.	.	1.9	0.3	12.2	0.0	.	.	2.5	0.3	16.1	12.5	1.5	57.5	0.0	.	.
Sweetened Dairy Products	46.7	29.6	64.5	44.5	35.8	53.6	64.3	36.5	84.9	43.0	34.9	51.5	37.5	17.4	63.1	45.9	37.6	54.4
Salty Snacks	40.0	8.2	83.2	94.2	87.6	97.4	75.0	48.1	90.7	94.6	87.5	97.7	80.0	43.7	95.4	92.9	85.7	96.6
Cookies	92.0	72.3	98.1	92.5	87.9	95.4	92.6	74.1	98.2	92.4	87.8	95.4	75.0	35.0	94.4	93.1	88.8	95.8
Canned Vegetables	50.0	1.9	98.1	86.1	78.2	91.5	100.0	.	.	85.2	77.1	90.7	75.0	17.9	97.6	85.8	77.8	91.3
Oils and Fats	28.6	6.3	70.3	14.1	8.5	22.5	0.0	.	.	15.5	9.7	23.9	33.3	2.5	90.5	14.6	9.0	22.8
Sauces and Dressings	40.0	8.2	83.2	85.2	80.3	89.1	85.7	55.7	96.6	84.3	79.2	88.3	66.7	42.1	84.6	85.7	80.7	89.5
Coffee and Tea	0.0	0.0	0.0	0.0	.	.	0.0	.	.	0.0	.	.	0.0	.	.	0.0	.	.
Candies and Desserts	73.3	45.6	90.0	71.7	66.8	76.2	73.3	54.6	86.3	71.6	66.6	76.2	60.0	33.9	81.4	72.3	67.4	76.7
Cereals, Beans, Other Grain Products	0.0	.	.	12.4	8.5	17.8	4.3	0.6	26.3	11.6	7.9	16.7	6.3	0.8	35.2	11.2	7.6	16.2
Packaged Fruits and Vegetables	0.0	.	.	0.0	.	.	0.0	.	.	0.0	.	.	0.0	.	.	0.0	.	.
Meat, Poultry, Seafood, and Egg	0.0	.	.	0.0	.	.	0.0	.	.	0.0	.	.	0.0	.	.	0.0	.	.
Sugar and Nonnutritive Sweeteners	40.0	8.2	83.2	37.5	22.5	55.4	0.0	.	.	38.9	24.4	55.7	50.0	1.9	98.1	37.1	22.8	54.2
Processed Meats	100.0	.	.	92.6	88.5	95.3	100.0	.	.	92.9	89.1	95.5	93.8	64.8	99.2	92.9	88.9	95.6
Juices	0.0	.	.	14.9	7.2	28.3	0.0	.	.	13.5	6.5	25.8	6.7	1.6	23.6	21.7	9.2	43.4
Nectars	40.0	8.2	83.2	24.4	14.0	39.2	0.0	.	.	26.0	15.6	40.0	26.7	13.7	45.4	25.0	10.6	48.5
Fruit-Flavored Drinks	81.8	47.3	95.8	76.9	63.5	86.5	0.0	.	.	77.8	65.8	86.4	12.5	1.5	57.5	87.3	75.5	93.9
Soda	0.0	.	.	57.1	40.3	72.5	0.0	.	.	57.1	40.3	72.5	100.0	.	.	54.5	37.4	70.6
Other Beverages	61.8	44.4	76.6	72.1	56.8	83.5	53.8	27.2	78.5	70.3	58.0	80.3	50.0	27.8	72.2	72.9	60.1	82.7
Nuts and Seeds	0.0	.	.	31.8	15.7	53.9	0.0	.	.	31.8	15.7	53.9	0.0	0.0	0.0	31.8	15.7	53.9
Cheese	100.0	.	.	90.4	85.1	94.0	83.3	50.4	96.1	91.1	85.7	94.6	75.0	17.9	97.6	90.9	85.7	94.4
Fruit Preserve	20.0	2.1	74.5	13.0	8.2	19.9	54.5	25.6	80.7	9.6	5.5	16.2	29.4	12.4	55.1	10.9	6.4	17.9
Total	50.7	45.0	56.4	57.9	56.1	59.6	63.0	56.8	68.9	56.8	55.1	58.5	43.5	38.3	48.9	58.7	57.0	60.4

Abbreviation: CI, confidence interval.

**Table 4:** Foods and beverages high in critical nutrients by the presence of the subcategories of nutrition claims in the front of the package.

Food Category	Claims for Nutrients of Concern ^1^						Claims for Nutrients not of Concern ^2^					
	Yes			No			Yes			No		
	%	95% CI		%	95% CI		%	95% CI		%	95% CI	
Breakfast Cereals and Granola Bars	64.7	47.3	79.0	57.4	44.7	69.2	48.0	34.5	61.8	73.3	58.5	84.3
Bakery Products	96.0	85.1	99.0	85.6	78.3	90.8	95.0	81.8	98.8	86.7	79.8	91.5
Convenience Foods	87.1	69.8	95.2	89.4	84.4	92.9	84.0	63.7	94.0	89.7	84.8	93.1
Unsweetened Dairy Products	33.3	2.6	90.5	0.0	0.0	0.0	7.7	1.0	41.2	0.0	.	.
Sweetened Dairy Products	88.9	46.7	98.6	42.1	34.2	50.5	81.8	59.7	93.2	38.6	30.5	47.4
Salty Snacks	85.0	61.6	95.2	93.2	85.6	96.9	70.0	35.8	90.7	93.9	87.0	97.2
Cookies	91.7	79.6	96.9	92.7	87.7	95.7	100.0	.	.	91.0	86.0	94.3
Canned Vegetables	50.0	1.9	98.1	86.1	78.2	91.5	100.0	.	.	85.3	77.3	90.8
Oils and Fats	61.1	37.1	80.7	5.7	2.4	13.0	10.0	1.2	49.7	15.6	9.6	24.4
Sauces and Dressings	85.7	63.1	95.5	84.2	79.1	88.3	100.0	.	.	84.3	79.3	88.2
Coffee and Tea	0.0	.	.	0.0	.	.	0.0	.	.	0.0	.	.
Candies and Desserts	92.0	80.3	97.0	68.6	63.2	73.5	79.5	63.8	89.5	70.9	65.7	75.5
Cereals, Beans, Other Grain Products	35.3	16.3	60.4	8.9	5.8	13.6	0.0	.	.	14.6	10.1	20.8
Packaged Fruits and Vegetables	0.0	.	.	0.0	.	.	0.0	.	.	0.0	.	.
Meat, Poultry, Seafood, and Egg	0.0	.	.	0.0	.	.	0.0	.	.	0.0	.	.
Sugar and Nonnutritive Sweeteners	75.0	17.9	97.6	33.3	19.3	51.1	0.0	.	.	37.8	23.7	54.4
Processed Meats	91.7	56.3	98.9	93.1	89.1	95.6	94.4	68.0	99.3	92.9	88.8	95.5
Juices	7.7	1.9	26.7	18.5	7.8	37.9	0.0	.	.	16.7	8.1	31.3
Nectars	58.3	29.6	82.3	15.8	7.2	31.2	27.3	12.5	49.6	25.0	12.2	44.3
Fruit-Flavored Drinks	100.0	.	.	70.2	55.6	81.6	86.1	70.4	94.2	66.7	46.9	81.9
Soda	100.0	.	.	34.8	18.1	56.2	66.7	9.5	97.4	56.3	38.7	72.3
Other Beverages	73.9	52.2	88.0	64.8	51.2	76.4	80.0	51.8	93.7	64.5	51.8	75.4
Nuts and Seeds	0.0	.	.	31.8	15.7	53.9	0.0	.	.	35.0	17.3	58.0
Cheese	0.0	.	.	91.6	86.5	94.9	100.0	.	.	90.0	84.5	93.7
Fruit Preserve	44.0	25.9	63.8	6.3	3.0	12.7	40.0	18.6	66.1	9.9	5.7	16.7
Total	74.0	69.7	77.9	54.8	53.0	56.6	57.4	52.8	61.9	57.2	55.5	59.0

Abbreviation: CI, confidence interval; ^1^ Includes nutrition claims for saturated fats, trans fats, sodium, and sugar; ^2^ Includes nutrition claims for vitamins, minerals, protein, and unsaturated fats.

## Supplementary Materials

The following are available online at https://www.mdpi.com/2072-6643/11/12/2967/s1, Table S1: Presence of claims in the front of the package of Brazilian packaged foods and beverages high in free sugars, saturated fat, and sodium. Brazil, 2017.
