# Supplementary material for: Conflicting Messages on Food and Beverage Packages: Front-of-Package Nutritional Labeling, Health and Nutrition Claims in Brazil
Source: Nutrients. 2019 Dec 5;11(12):2967. doi: 10.3390/nu11122967 (PMC6950677; doi:10.3390/nu11122967)
Supplement: Supplementary file 1 [file nutrients-11-02967-s001.pdf]

Table S1. Presence of claims in the front of the package of Brazilian packaged foods and beverages high in free sugars, saturated fat and sodium. Brazil, 2017.

Food Category	Presence of Health Claims						Presence of Nutrition Claims						Presence of Environment Claims					
	High in Free Sugars		High in Saturated Fat		High in Sodium		High in Free Sugars		High in Saturated Fat		High in Sodium		High in Free Sugars		High in Saturated Fat		High in Sodium	
	N	%	N	%	N	%	N	%	N	%	N	%	N	%	N	%	N	%
Breakfast cereals and granola bars	2	33.3	12	28.6	1	2.4	14	66.7	25	30.1	4	4.8	3	60.0	2	20.0	1	10.0
Bakery products	0	0.0	10	20.0	34	68.0	0	0.0	11	13.9	58	73.4	.	.	1	25.0	1	25.0
Convenience foods	.	.	18	32.1	45	80.4	0	0.0	16	28.1	47	82.5	0	0.0	1	7.1	12	85.7
Unsweetened dairy products	0	0.0	1	4.5	0	0.0	0	0.0	1	4.8	0	0.0	.	.	0	0.0	0	0.0
Sweetened dairy products	10	71.4	6	11.5	2	3.8	11	73.3	6	12.5	3	6.3	.	.	.	.	.	.
Salty snacks	0	0.0	13	52.0	12	48.0	0	0.0	14	45.2	17	54.8	.	.	7	100.0	6	85.7
Cookies	9	50.0	28	53.8	9	17.6	13	52.0	45	49.5	21	23.1	.	.	7	87.5	0	0.0
Canned vegetables	.	.	1	14.3	5	71.4	.	.	0	0.0	3	75.0	.	.	0	0.0	3	100.0
Oils and fats	0	0.0	4	30.8	1	8.3	0	0.0	9	27.3	5	15.2	.	.	0	0.0	0	0.0
Sauces and dressings	0	0.0	8	24.2	21	63.6	6	75.0	18	46.2	35	89.7	0	0.0	1	12.5	5	62.5
Coffee and tea	.	.	0	0.0	0	0.0	.	.	.	.	.	.	.	.	0	0.0	0	0.0
Candies and desserts	12	44.4	19	32.8	4	6.9	17	39.5	28	29.8	17	18.1	.	.	4	57.1	0	0.0
Cereals, beans, other grain products	0	0.0	0	0.0	2	3.8	0	0.0	1	1.1	9	10.3	0	0.0	0	0.0	1	5.9
Packaged fruits and vegetables	0	0.0	0	0.0	0	0.0	0	0.0	0	0.0	0	0.0	0	0.0	0	0.0	0	0.0
Meat, poultry, seafood, and egg	.	.	0	0.0	0	0.0	.	.	0	0.0	0	0.0	.	.	0	0.0	0	0.0

Sugar and other noncaloric sweeteners	.	.	0	0.0	0	0.0	0	0.0	0	0.0	0	0.0	.	.	0	0.0	0	0.0
Processed meats	.	.	14	43.8	29	90.6	.	.	12	40.0	28	93.3	.	.	0	0.0	2	100.0
Juices	2	15.4	0	0.0	0	0.0	3	21.4	0	0.0	0	0.0	3	75.0	0	0.0	0	0.0
Nectars	3	100.0	0	0.0	0	0.0	3	100.0	0	0.0	0	0.0	1	100.0	0	0.0	0	0.0
Fruit-flavored drinks	4	50.0	0	0.0	0	0.0	12	60.0	0	0.0	0	0.0	.	.	.	.	.	.
Sodas	0	0.0	0	0.0	0	0.0	3	25.0	0	0.0	0	0.0	.	.	.	.	.	.
Other beverages	11	47.8	1	1.9	9	16.7	8	38.1	1	2.0	6	12.0	2	33.3	0	0.0	0	0.0
Nuts and seeds	.	.	.	.	.	.	.	.	0	0.0	0	0.0	.	.	0	0.0	0	0.0
Cheeses	.	.	1	5.9	14	82.4	.	.	20	50.0	36	90.0	.	.	0	0.0	2	100.0
Fruit preserve	3	60.0	0	0.0	0	0.0	5	71.4	0	0.0	0	0.0	0	0.0	0	0.0	0	0.0
Total	56	38.6	136	17.8	188	24.5	95	44.4	207	20.9	289	29.2	9	37.5	23	13.6	33	18.2
